# Gab2 Promotes Hematopoietic Stem Cell Maintenance and Self-Renewal Synergistically with STAT5

**DOI:** 10.1371/journal.pone.0009152

**Published:** 2010-02-10

**Authors:** Geqiang Li, Zhengqi Wang, Kristy L. Miskimen, Yi Zhang, William Tse, Kevin D. Bunting

**Affiliations:** 1 Division of Hematology-Oncology, Department of Medicine, Case Western Reserve University, Cleveland, Ohio, United States of America; 2 Center for Stem Cell and Regenerative Medicine, Cleveland, Ohio, United States of America; 3 Case Comprehensive Cancer Center, Cleveland, Ohio, United States of America; Oklahoma Medical Research Foundation, United States of America

## Abstract

**Background:**

Grb2-associated binding (Gab) adapter proteins play major roles in coordinating signaling downstream of hematopoietic cytokine receptors. In hematopoietic cells, Gab2 can modulate phosphatidylinositol–3 kinase and mitogen associated protein kinase activities and regulate the long-term multilineage competitive repopulating activity of hematopoietic stem cells (HSCs). Gab2 may also act in a linear pathway upstream or downstream of signal transducer and activator of transcription-5 (STAT5), a major positive regulator of HSC function. Therefore, we aimed to determine whether Gab2 and STAT5 function in hematopoiesis in a redundant or non-redundant manner.

**Methodology/Principal Findings:**

To do this we generated Gab2 mutant mice with heterozygous and homozygous deletions of STAT5. In heterozygous STAT5 mutant mice, deficiencies in HSC/multipotent progenitors were reflected by decreased long-term repopulating activity. This reduction in repopulation function was mirrored in the reduced growth response to early-acting cytokines from sorted double mutant c-Kit^+^Lin^−^Sca-1^+^ (KLS) cells. Importantly, in non-ablated newborn mice, the host steady-state engraftment ability was impaired by loss of Gab2 in heterozygous STAT5 mutant background. Fetal liver cells isolated from homozygous STAT5 mutant mice lacking Gab2 showed significant reduction in HSC number (KLS CD150^+^CD48^−^), reduced HSC survival, and dramatic loss of self-renewal potential as measured by serial transplantation.

**Conclusions/Significance:**

These data demonstrate new functions for Gab2 in hematopoiesis in a manner that is non-redundant with STAT5. Furthermore, important synergy between STAT5 and Gab2 was observed in HSC self-renewal, which might be exploited to optimize stem cell-based therapeutics.

## Introduction

Grb2-associated binding protein-2 (Gab2) is tyrosine phosphorylated by several early acting cytokine receptors such as Flt3, c-Kit, interleukin (IL)-3R, and c-Mpl and contains binding sites for SH2 and SH3 domains that promote binding to signaling molecules[1]–[3]. Gab2 activates the phosphatidylinositol-3′-kinase (PI3-K) and the mitogen activated protein kinase (MAPK) pathways and can regulate hematopoietic cell survival, proliferation, cytoskeleton reorganization, and adhesion/migration functions[2], [4]–[7]. The original cloning and description of Gab2 showed that a Gab2 mutant lacking amino acids 604–662 impaired IL-3 induced signal transducer and activator of transcription-5 (STAT5) activation in BaF3 cells, indicating that Gab2 may act upstream of STAT5[1]. We have previously identified Gab2 as located on a chromosome 7 STAT5 modifier locus, containing several hundred genes, that modulates hematopoietic stem cell (HSC) engraftment during steady-state hematopoiesis[8]. STAT5 is a latent transcription factor that can be activated by phosphorylation by Janus kinases (JAKs) in the cytoplasm, leading to dimerization, DNA binding, and retention within the nucleus[9]. STAT5 is a major regulator of hematopoietic development in multiple hematopoietic lineages and is essential for HSC “fitness” as characterized by competitive repopulation of lethally-irradiated hosts[10], [11].

Cross-talk between JAK/STAT5 and PI3-K pathways has been described in hematopoietic and non-hematopoietic cells. Interaction between these pathways is important for pro-survival signaling[12] in neural cells. Several reports in IL-2 responsive hematopoietic cell lines also suggest that STAT5 and PI3-K play important roles in cytokine responsiveness. In response to IL-2, a delayed cycloheximide-sensitive mechanism for coordinated cyclin D2 expression involves both PI3-K and STAT5 activation[13]. Interestingly, knockdown of STAT5 impaired IL-2 induced Shc mediated stimulation of Akt activation through the IL-2 receptor[14] suggesting that Gab2 might function downstream of STAT5. Another study showed that mutation of the SH2-containing phosphatase (SHP-2) binding sites of Gab2 influenced STAT5 activation and proliferation in response to IL-2[15]. Since STAT5 is a substrate for SHP-2 phosphatase[16], this study demonstrates another unique manner in which Gab2 might function upstream of STAT5.

STAT5 and PI3-K activation is also observed downstream of thrombopoietin (TPO)/c-Mpl signaling to regulate the expression level of the direct STAT5 target gene Bcl-X_L_ [17]. TPO/c-Mpl signaling is critically important for HSC repopulation, self-renewal, and interaction with the HSC niche[18]–[21]. Enhanced sensitivity to inhibition of STAT5, SHP-2, and Gab2[22] has also been described in Bcr/Abl-induced oncogenic activities. Based on these lines of evidence, we initially hypothesized that Gab2 may depend entirely on STAT5 in HSC to regulate important functions such as survival and self-renewal. Since all prior work in this area has been done in cultured cell lines, it was important to analyze mouse models of STAT5 and Gab2. To date, work with single Gab2 or STAT5 knockout mice has shown similar phenotypes in mast cells, where STAT5 and Gab2 are involved in IL-3/SCF signaling[4], [23]–[25]. STAT5 was much more critical for early hematopoiesis including HSC self-renewal[10], whereas Gab2 was active in multilineage hematopoietic repopulation but was not essential for serial transplantation ability[26].

Considering that Gab2 promotes the PI3-K and Erk pathways and may act upstream or downstream of STAT5, we set out to determine whether STAT5 and Gab2 function in a redundant or non-redundant manner to control HSC function. The relationship between STAT5- and Gab2-mediated signals in early hematopoiesis was explored at the genetic level through generation and characterization of compound mutant mice. We studied hematopoiesis at various stages of development as permitted by the limiting survival of STAT5ab^null/null^ mice and found that Gab2 has functions that are non-redundant with STAT5 in controlling HSC survival and self-renewal.

## Materials and Methods

### Mice, Fetal Liver Isolation and Genotyping

STAT5ab^+/null^ mice were obtained from Lothar Hennighausen (NIDDK, NIH)[27]. Gab2^−/−^ mice were obtained from Toshio Hirano (Osaka University)[4]. The C57BL/6 (CD45.2) mice and the congenic B6.SJL-Ptprc^a^Pep3^b^/BoyJ (CD45.1) strain were obtained from the Jackson Laboratory (Bar Harbor, ME). All mutant mice were on the C57BL/6 background and housed in a specific pathogen-free environment. All mouse studies were approved by the Institutional Animal Care and Use Committee at Case Western Reserve University. Gab2^+/−^STAT5ab^+/null^ mice were inter-crossed to yield Gab2^−/−^STAT5ab^null/null^ double knockout embryos. Fetal liver (FL) cells from these embryos were collected at embryonic day 14.5 (E14.5) and genotyped the same day by PCR. The following primers were used for STAT5 genotyping: 5′-AGC AGC AAC CAG AGG ACT AC-3′, 5′-GAA AGC ATG AAA GGG TTG GAG-3′ and 5′-CCC ATT ATC ACC TTC TTT ACA G-3′. The following primers were used for Gab2 genotyping: 5′-AAT GTA GAC AGT CAG TGC CTA GAG GGT CCA-3′; 5′-CAT GTA TCA TGA CAT TTG TGC TCC AAC A-3′, and 5′-CAG CAG CCT CTG TTC CAC ATA CAC TTC AT-3′. The PCR parameters for genotyping fetal liver DNA were: 94°C, 30 sec.; 55°C, 30 sec.; 72°C, 1 min. for 35 cycles.

### Mouse Peripheral Blood Hematology

Peripheral blood was obtained following puncture of the retroorbital venous sinus using a microcapillary tube. For red and white blood cell counts, cells were diluted in isotonic saline solution and analyzed using a Coulter counter (Beckman Coulter, Inc., Fullerton, CA).

### Colony-Forming Unit Assay

Total CFU-C colonies were assayed by standard methods according to the Stem Cell Technologies manual. Briefly, either fetal liver or BM cells were plated in EPO-containing MethoCult GF M3334 methylcellulose medium (Stem Cell Technologies, Vancouver, BC, Canada) with added recombinant murine IL-3 (20 ng/ml), recombinant human IL-6 (50 ng/ml), recombinant murine stem cell factor (SCF; 50 ng/ml). On day 7 of culture, colonies of greater than 50 cells were counted and the frequency of total CFU-C per 1×10^5^ cells was calculated based on the input number of BM cells. All hematopoietic cytokines were from R&D Systems (Minneapolis, MN).

### Flow Cytometric Determination of BM and Fetal Liver HSC Numbers

Fetal liver or BM cells were harvested in PBS/2% FBS (Hyclone). For BM stem cell staining, after erythrocyte lysis, PE-conjugated lineage markers, Ly-6G (Gr-1), CD11b (Mac-1), CD45R/B220, CD4 (L3T4), CD8 (Ly2), and Ter119/Ly76 were used. The cells also were stained with antibodies to fluorescein isothiocyanate (FITC)-conjugated Ly-6A/E (Sca-1) and to APC-conjugated CD117 (c-Kit). Anti Flk2 conjugated to Pacific Blue (PB) was used to define short-term repopulating HSC (KLS Flk2^+^) and long-term repopulating HSCs (KLS Flk2^neg^). After antibody staining, 7-amino-actinomycin D (7-AAD) at 2 µg/ml was added into cells and incubated for 20 min immediately before flow cytometry to exclude dead cells. Staining for fetal liver stem cells was slightly different. PE-conjugated lineage markers were the same for BM cell staining except that CD11b (Mac-1) was not included. The fetal liver cells were also stained with c-Kit-APC and Sca-1-FITC and in some analyses with CD150-APC-Cy7 and CD48-PB. All flow cytometry was performed on a BD LSRII with 4 lasers (355 nM solid state UV laser, 405 nM solid state violet laser, 488 nM solid state blue laser, 633 nM red HeNe laser) (Becton Dickinson, Franklin Lakes, NJ). The data were analyzed using the FlowJo software program (Tree Star, Ashland, OR).

### Flow Cytometric Assay for FL HSC Survival

To analyze cell death in FL HSC, E14.5 FL cells were counted and stained with biotin-conjugated antibodies to the lineage markers Ly-6G (Gr-1), CD45R/B220, CD4 (L3T4), CD8 (Ly2), and Ter119/Ly76. Cells were incubated with anti-biotin microbeads (Miltenyi Biotec), followed by AutoMACS depletion through a fresh separation column. The negative pass-through fraction (LN^−^) was counted and 5×10^5^ cells were stained with the above lineage markers and anti-c-Kit-APC and anti-Sca-1-PE-Cy7. The lineage markers were detected by a secondary streptavidin-APC-Cy7 step. Finally, the cells were stained with Annexin V-PE (BD Bioscience) and DAPI (Invitrogen) according to the manufacturer's instructions.

### Long-Term Repopulation and Secondary Transplantation

For fetal liver non-competitive transplantation, 8- to 12-week-old male B6.SJL-Ptprc^a^Pep3^b^/BoyJ mice were lethally irradiated with 11 Gy using γ^137^Cs Shepherd Mark I irradiator (JL Shepherd, San Fernando, CA). The entire population of E14.5 fetal liver cells was resuspended thoroughly in PBS/2% FBS) and transplanted into 5 recipient mice by intravenous injection through the lateral tail vein. Donor engraftment was monitored 8–16 weeks after transplantation by staining with antibodies to CD45.2 and to individual lineage markers that included CD4, B220, Gr-1, or Ter119. Peripheral blood cells were counted by Coulter counter as described above.

### Competitive Repopulation Assays

For BM competitive transplantation, BM cells from CD45.2 donor mice were mixed with CD45.1 competitor mouse BM cells at a 1∶1 ratio. The mixed cells were then transplanted into lethally irradiated CD45.1 B6.SJL-Ptprc^a^Pep3^b^/BoyJ recipient mice (1100 rads). Donor engraftment was monitored 8–16 weeks after transplantation by staining with antibody to CD45.2 and antibodies to lineage-specific antigens. For newborn injection experiments, 2–3 day old newborn pups were injected intraperitoneally with donor adult BM cells exactly as described previously[8].

### Statistical Analyses

For multiple comparisons, two-way ANOVA analysis was used. We also use either one-way ANOVA, or Wilcoxon-Mann-Whitney U tests for wild-type vs. mutant single comparisons. *P* values of less than 0.05 were considered statistically significant. All statistics were performed using SPSS16.0 (SPSS, Inc.) or StatistiXL 1.8, which is designed and written by Alan Roberts and Philip Withers (University of Western Australia., Australia). Student's two-tailed T test was performed using InStat 1.5 (University of Reading, UK).

## Results

### Cell Intrinsic Functional Defects in Gab2^−/−^STAT5ab^+/null^ HSC

To assess whether STAT5 and Gab2 were redundant during normal hematopoiesis, compound mutant mice with targeted alleles of STAT5 and Gab2 were generated and characterized at various developmental stages. Comparisons between adult STAT5ab^+/null^ and Gab2^−/−^ genotype was the first measure tested, since both Gab2^−/−^ and STAT5ab^+/null^ mice and their Gab2^−/−^STAT5ab^+/null^ cross were born at normal Mendelian ratio and had no apparent defects in survival long-term. No evidence of Gab2-mediated defects in peripheral blood hematology were observed, similar to our prior study, and Gab2 deletion did not decrease blood counts in STAT5ab^+/null^ mice (**Fig. S1A**). Since the peripheral blood counts were unaffected by the double mutant, we focused on the BM compartment and determined that the absolute number of LT-HSC were normal in all single and double mutant mice (**Fig. S1B**).

**Figure 1:**
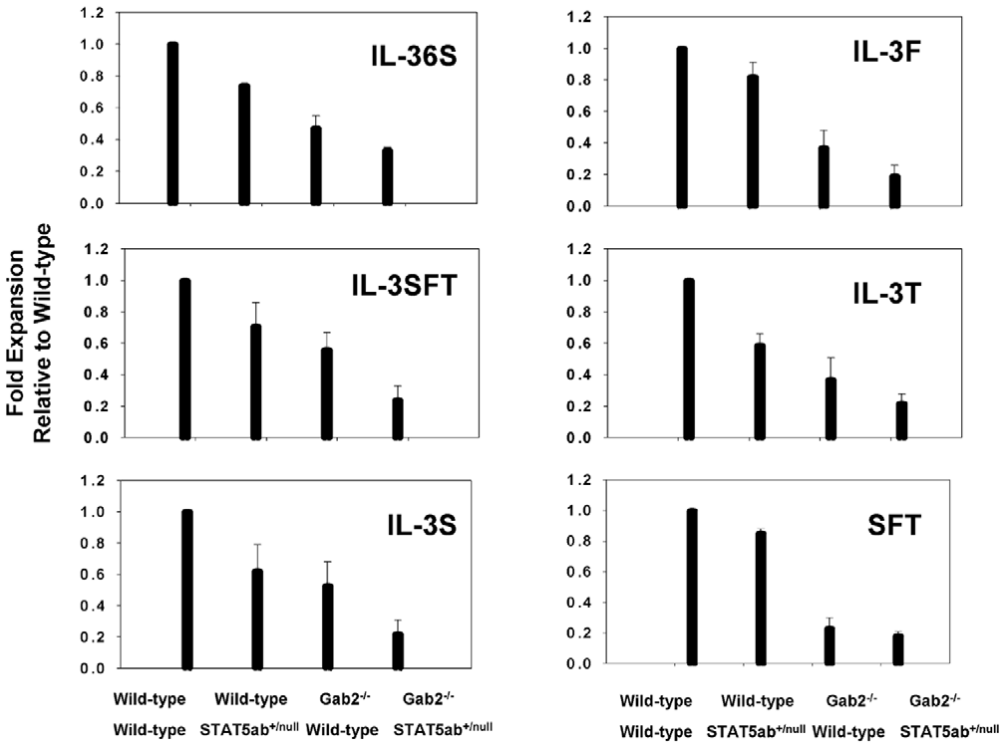
Combined deficiency of Gab2 with heterozygous STAT5 reduces KLS cell responses to early acting cytokines. KLS cells were sorted by flow cytometry and plated in 96-well plates in the presence of various cytokine cocktails. Total nucleated cells were counted 6 days later. IL-3 indicates murine IL-3 (20 ng/ml); 6, human IL-6 (50 ng/ml); S, murine stem cell factor (50 ng/ml); F, murine Flt3-ligand (50 ng/ml); and T, murine thrombopoietin (50 ng/ml). The cell expansion in each group of cytokines was plotted relative to wild-type control cells for each stimulation experiment. The results are the average ± SD for 3 experiments. Absolute cell expansions were comparable to previous descriptions[26], [28].

**Figure 2:**
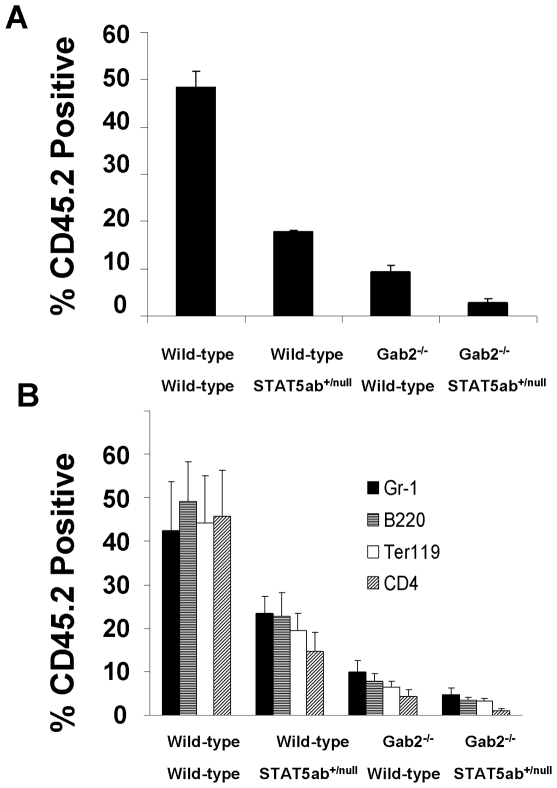
Combined deficiency of Gab2 with heterozygous STAT5 leads to declines in adult HSC activity. **A.** BM cells from 3–5 donor mice were collected and mixed 1:1 with wild-type competitor (CD45.1) and transplanted into lethally-irradiated hosts for competitive repopulation analysis. Recipient mice were bled 12 weeks later for flow cytometry analysis. Shown is the average of two independent experiments with 5 mice per group in each experiment. **B.** At 16 weeks post transplant, mice were bled again for multilineage analysis for the same two independent experiments. Peripheral blood leukocytes were stained with antibodies to multiple lineage markers including Gr-1, B220, Ter119, and CD4.

Since peripheral blood counts and steady-state BM cellularity do not provide indicators of stress hematopoiesis, we next tested KLS cytokine stimulation which measures a combination of growth and differentiation response, which differs significantly from maintenance of the steady-state HSC pool *in vivo*. KLS cells were sorted into 96 well plates and stimulated with cytokines as previously described[26], [28] (**Fig. 1**). Significant declines in cytokine response were observed for deficiency of STAT5 or Gab2 alone, as well as further decreases in Gab2^−/−^STAT5ab^+/null^ KLS cells relative to single mutants in cocktails containing IL-3, IL-6, and SCF (Gab2^−/−^STAT5ab^+/null^ vs. Gab2^−/−^, P = 0.03; Gab2^−/−^STAT5ab^+/null^ vs. STAT5ab^+/null^, P<0.01, t-test) or with IL-3, SCF, Flt-3 ligand, and TPO (Gab2^−/−^STAT5ab^+/null^ vs. Gab2^−/−^, P = 0.02; Gab2^−/−^STAT5ab^+/null^ vs. STAT5ab^+/null^, P<0.01, t-test). Notably these cytokine combinations induce the strongest proliferation and differentiation from purified KLS cells. To determine whether defects were also observed *in vivo*, BM cells were harvested and transplantation experiments performed. Two independent competitive repopulation experiments were performed using adult BM cells with the same results. The average results of the two transplantation experiments are shown in **Fig. 2A**. STAT5ab^+/null^ and Gab2^−/−^ BM had competitive multilineage HSC repopulating defects averaging 36.7±6.4% and 17.5±3.5% of wild-type respectively. Notably, Gab2^−/−^STAT5ab^+/null^ BM cells had an average 9.5±1.7% of wild-type engraftment. The percentage of donor engraftment between single mutant (Gab2^−/−^ or STAT5ab^+/null^) and Gab2^−/−^STAT5ab^+/null^ were significantly different (P<0.001 for STAT5ab^+/null^Gab2^−/−^ vs. STAT5ab^+/null^ or Gab2^−/−^STAT5ab^+/null^ vs. Gab2^−/−^, Mann-Whitney U test). The reduction of donor engraftment in Gab2^−/−^STAT5ab^+/null^ mice was significant (P<0.001, 2-way ANOVA). Further multilineage analysis showed that the long-term reductions in repopulation occurred in a primitive cell type (**Fig. 2B**). STAT5ab^+/null^ and Gab2^−/−^ mice were significantly different for all lineages (P = 0.001 for Gr-1 and P<0.001 for all others; 2-way ANOVA).

### STAT5 and Gab2 Promote Long-Term Multilineage Engraftment in Non-Ablated Newborn Mice

**Figure 3:**
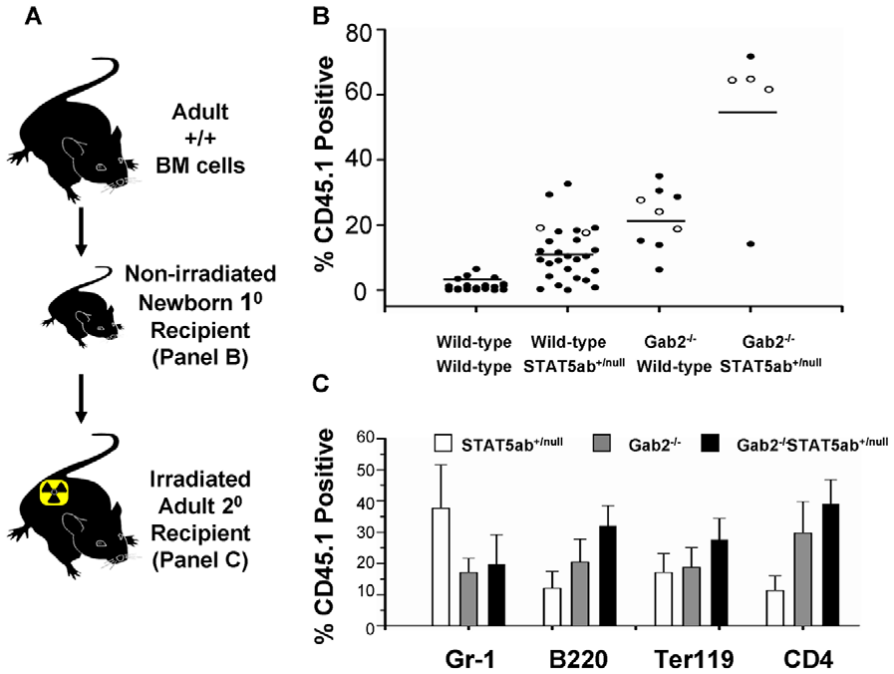
Combined deficiency of Gab2 with heterozygous STAT5 allows replacement of newborn HSC with a donor graft. **A.** Pups generated from inter-cross of Gab2^+/−^STAT5ab^+/null^ mice were injected intraperitoneally with 1×10^7^ CD45.1 positive BM cells 2–3 days after birth. At 4 weeks of age, the newly weaned mice were genotyped for STAT5 and Gab2. From multiple sets of experiments, totals of WT N = 22; STAT5ab^+/null^ N = 27; Gab2^−/−^ N = 9; and Gab2^−/−^STAT5ab^+/null^ N = 5 mice were obtained and analyzed for evidence of long-term donor engraftment. **B.** At 16 weeks following injection, the mice were analyzed by flow cytometry of the peripheral blood leukocytes for engraftment with CD45.1 positive donor cells. Horizontal bars indicate the average for all mice analyzed per group. All comparisons were done by Mann-Whitney U test. **C.** BM was pooled from primary recipients and injected into 5 lethally-irradiated secondary recipients. Individual primary donors utilized for secondary transplantation are marked in panel B by open circles. The percentage of CD45.1 positive cells co-staining for Gr-1, B220, Ter119, or CD4 is shown from secondary transplantation and analysis 12 weeks later. The wild-type group was not included in the secondary transplantation since the primary recipients had little to no evidence of donor engraftment.

Non-ablated engraftment of newborn mice is a very stringent test for HSC function. In these transplants, we found that STAT5ab^+/null^ hosts could be significantly engrafted on average 8-fold higher when injected with 1×10^7^ BM cells as newborn pups (**Fig. 3A**) (P<0.01, Mann-Whitney U test). In contrast, wild-type mice showed very low levels of engraftment. As expected, no STAT5ab^null/null^ mice were obtained due to perinatal lethality. Mice lacking Gab2 expression could also be engrafted significantly at 16-fold higher levels than wild-type control mice (P<0.01, Mann-Whitney U test), with levels similar to those of STAT5 haploinsufficiency. Strikingly, deletion of Gab2 combined with STAT5 haploinsufficiency led to a synergistic 40-fold rise in long-term engraftment levels averaging 55±23%. The combination of STAT5ab^+/−^ and Gab2^−/−^ genotype allowed significantly increased donor engraftment (P<0.001; 2-way ANOVA). Differences between single mutants and Gab2^−/−^STAT5ab^+/null^ were significant (Gab2^−/−^ vs. Gab2^−/−^STAT5ab^+/null^, P = 0.042 and STAT5ab^+/null^ vs. Gab2^−/−^STAT5ab^+/null^, P = 0.001; Mann-Whitney U test). Multilineage donor engraftment was observed in lethally-irradiated recipients of secondary transplantation (**Fig. 3B**). No significant differences were observed in secondary reconstitution (1-way ANOVA), except there was a modest significant difference between Gab2^−/−^ and Gab2^−/−^STAT5ab^+/null^ in B220^+^ cells (Mann-Whitney U test).

### Decreased Fetal Liver CFU-C, HSC Number, and HSC Survival in the Complete Absence of Both STAT5 and Gab2

**Figure 4:**
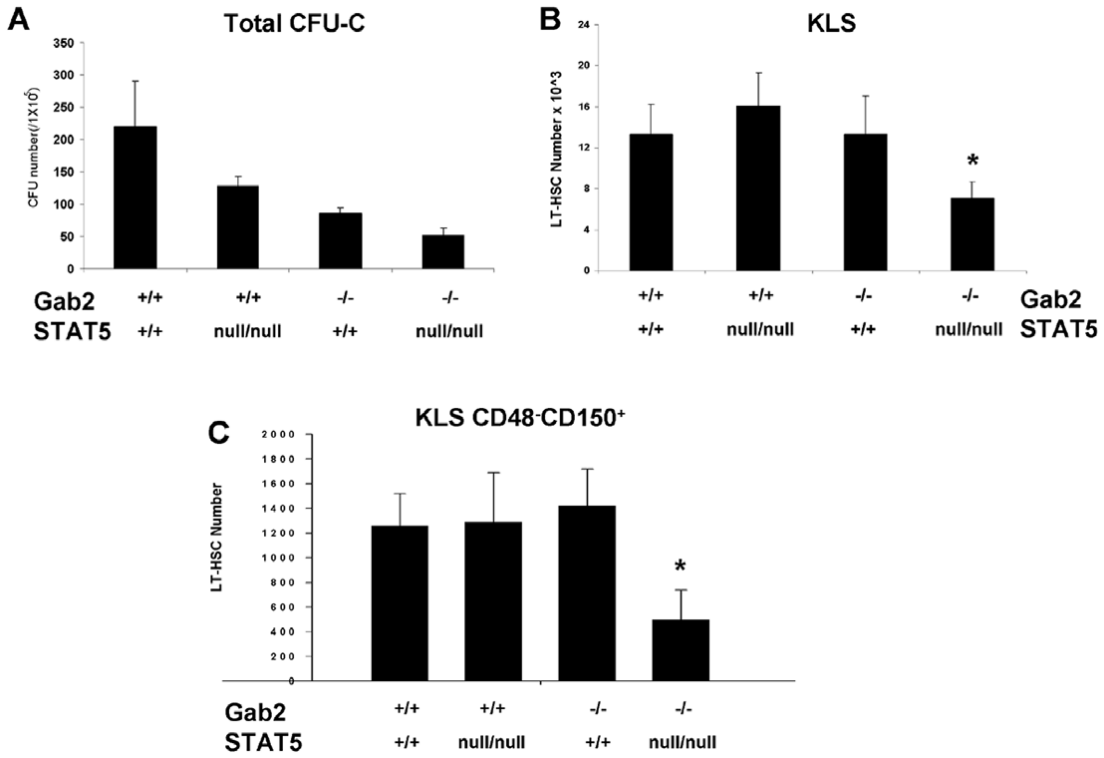
STAT5 and Gab2 double mutant fetal liver has reduced CFU-C and HSC. Gab2^−/−^STAT5ab^null/null^ and littermate control mice derived from Gab2^+/−^STAT5ab^+/null^ inter-cross were generated and characterized. **A.** Fetal liver cells were plated into methylcellulose medium for Wild-type N = 3; STAT5ab^null/null^ N = 3; Gab2^−/−^ N = 3; STAT5ab^null/null^Gab2^−/−^ N = 4. All 4 groups were significantly different from each other (P<0.005, Student's two-tailed t-test). **B.** The HSC enriched c-Kit^+^Lin^−^Sca-1^+^ (KLS) population per fetal liver was determined for Wild-type N = 3; STAT5ab^null/null^ N = 4; Gab2^−/−^ N = 6; STAT5ab^null/null^Gab2^−/−^ N = 5. **C.** The average number of long-term HSC (KLS CD150^+^CD48^−^) cells per FL were determined for Wild-type N = 3; STAT5ab^null/null^ N = 6; Gab2^−/−^ N = 4; Gab2^−/−^STAT5ab^null/null^ N = 5.

Since STAT5ab^null/null^ mice were not alive at the newborn or adult stages of development, we analyzed E14.5 fetal liver (FL) where STAT5ab^null/null^ embryos can survive[29]. At E14.5, the expected Mendelian ratio (1/16) of double mutant was obtained (15/231), indicating the STAT5/Gab2 double mutant embryos have no overall survival disadvantage at this stage of development. However, the CFU-C frequency of STAT5ab^null/null^Gab2^−/−^ FL was reduced to 24% of normal (N = 4) compared with STAT5ab^null/null^ or Gab2^−/−^ FL whose CFU-C frequency were reduced to 58% and 39% of wild-type respectively (**Fig. 4A**). The Gab2^−/−^STAT5ab^null/null^ CFU-C frequency was significantly lower than either Gab2^−/−^ or STAT5ab^null/null^ groups (P<0.006, Mann-Whitney U Test; P = 0.014, 2-way ANOVA). The specific types of myeloid CFU-C were not enumerated for these studies since prior work showed that STAT5 and Gab2 mutant mice have reduced numbers of all types with no particular bias toward a single lineage.

Since the number of CFU-C was most reduced in double mutant FL cells, we examined more primitive HSC populations by flow cytometry for the frequency of HSC. The KLS and the LT-HSC fraction (KLS CD150^+^CD48^−^) population were found to be present at normal number in FL cells lacking either STAT5 or Gab2. Gating for each of these populations is shown in **Fig. S2**. However, when both STAT5 and Gab2 were deleted, the absolute number of total HSC and LT-HSC was significantly reduced by 2- to 3-fold relative to wild-type (**Fig. 4B, 4C**). Differences between single mutant (Gab2^−/−^ or STAT5ab^null/null^) and Gab2^−/−^STAT5ab^null/null^ were significant (P<0.001; Mann-Whitney U test). The reduction of KLS cells (P = 0.009; 2-way ANOVA) and LT-HSC (P<0.001; 2-way ANOVA) in STAT5ab^null/null^ and Gab2^−/−^ was synergistic, suggesting that maintenance of the FL HSC pool depends upon both STAT5 and Gab2 together.

**Figure 5:**
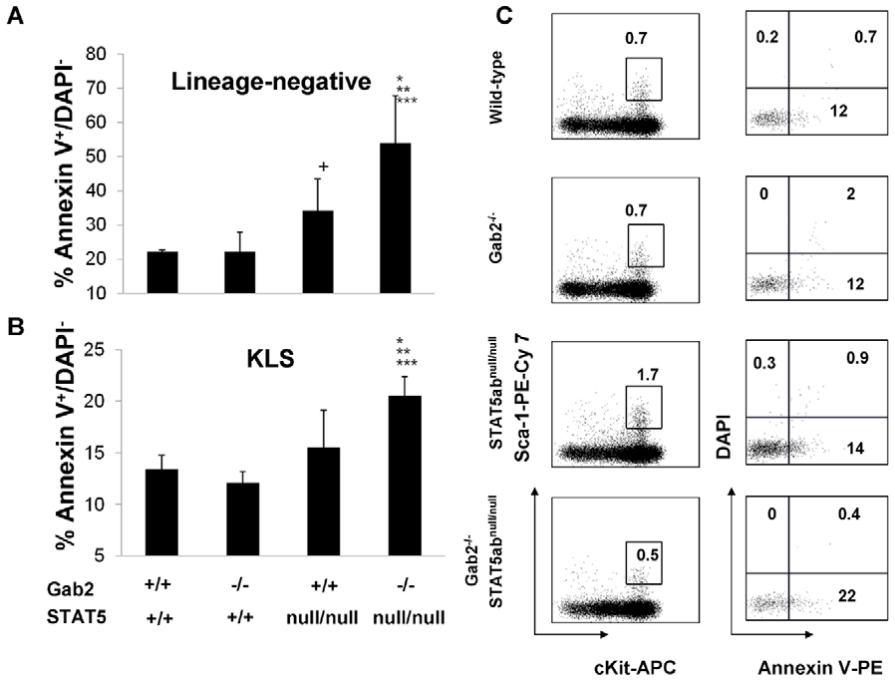
STAT5 and Gab2 double mutant fetal liver HSCs have reduced survival. Analysis of the frequency of early apoptotic cells by the Annexin V - DAPI flow cytometry assay on E14.5 FL cells. Early apoptotic cells were defined as Annexin V^+^/DAPI^−^. **A.** Percentages of apoptosis in the lineage-negative fraction. **B.** Percentages of apoptosis in the KLS fraction. (*: Gab2^−/−^STAT5ab^null/null^ vs. STAT5ab^null/null^ p<0.05; **: Gab2^−/−^STAT5ab^null/null^ vs. Gab2^−/−^ P<0.05; ***: Gab2^−/−^STAT5ab^null/null^ vs. Wild-type P<0.05; +: Wild-type vs. STAT5ab^null/null^ p<0.05). For panels A and B, the numbers of mice in each of the 4 groups were as follows: Wild-type N = 4; STAT5ab^null/null^ N = 7; Gab2^−/−^ N = 3; STAT5ab^null/null^Gab2^−/−^ N = 4. **C.** A representative flow cytometry plot showing Annexin V and DAPI gating on KLS cells among the 4 groups.

To better understand why KLS cell numbers were reduced, FL lineage-negative and KLS populations were analyzed by flow cytometry to determine the fraction of Annexin V stained cells. A marked increase in early apoptosis as measured by increased Annexin V^+^DAPI^−^ cells was observed for double mutant FL cells relative to wild-type littermate control (**Fig. 5**). The difference was significantly higher than either STAT5 or Gab2 mutant alone. Significantly reduced cell survival was observed in the KLS fraction (P = 0.02; 2-way ANOVA), but not in the LIN- fraction (P = 0.056; 2-way ANOVA), indicating non-redundant function in regulating survival occurred in primitive HSC, a finding that is consistent with the reduced KLS number only in the STAT5/Gab2 double mutant mice.

### Synergistic Defects in Self-Renewal of Fetal Liver HSC Completely Lacking Both STAT5 and Gab2

**Figure 6:**
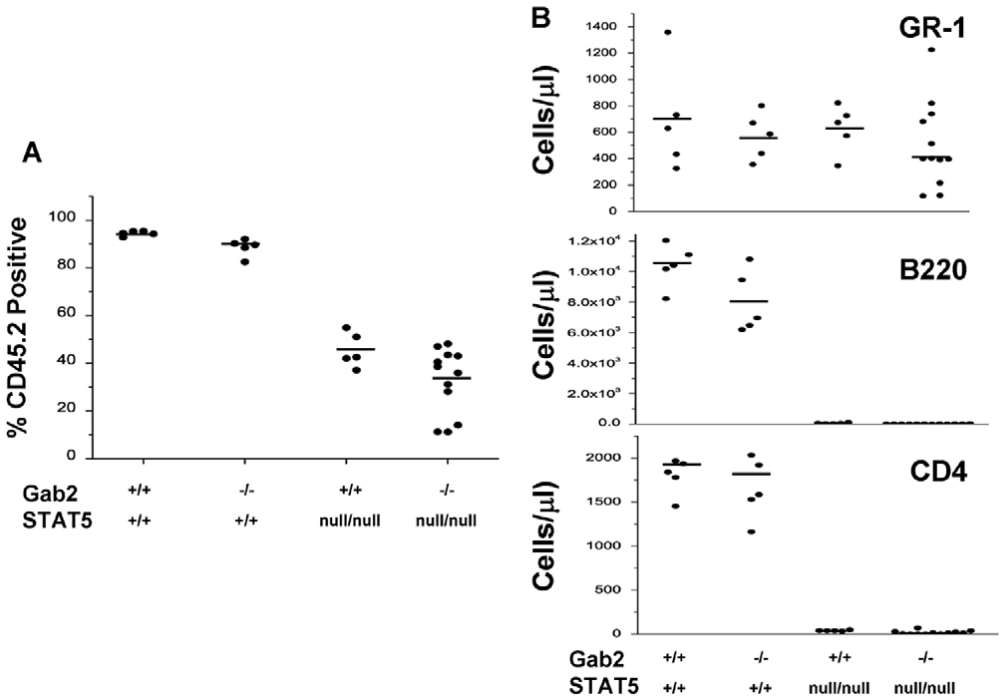
STAT5 and Gab2 double mutant fetal liver HSCs partially repopulate the myeloid lineage of primary recipients. Gab2^−/−^STAT5ab^null/null^ and littermate control embryos were used to derive FL cells. FL cells from 3 independent Gab2^−/−^STAT5ab^null/null^ embryos were each injected into 5 lethally-irradiated CD45.1 positive hosts to determine the hematopoietic repopulating potential. For other groups, 1 FL was injected into 5 recipient mice. **A.** The percentage of donor engraftment was determined by flow cytometry for expression of CD45.2. **B.** Peripheral lympho-myeloid blood donor contribution was determined by flow cytometry for Gr-1, B220, or CD4, 16 weeks later for three independent transplant experiments. The total number of mice from all 3 experiments was WT N = 5; Gab2^−/−^ N = 5; STAT5ab^null/null^ N = 5; Gab2^−/−^STAT5ab^null/null^ N = 12. Each dot represents an individual mouse that was analyzed. Horizontal bars indicate the average for all mice analyzed per group.

To determine whether double mutant FL HSC were capable of long-term engraftment of irradiated hosts, total FL cells from 3 independent embryos or different litters were transplanted into CD45.1 recipient mice at a donor to recipient ratio of 1 FL per 5 recipient mice. The percentage of donor CD45.2 engraftment was determined at times up to 16 weeks later (**Fig. 6A**). The donor engraftment was not significantly changed by combined STAT5ab^null/null^ and Gab2^−/−^ genotype (P = 0.415; 2-way ANOVA). Differences between STAT5ab^null/null^ and Gab2^−/−^STAT5ab^null/null^ were not significant (P = 0.104; Mann-Whitney U test). When analyzing the overall contribution of the donor to host hematopoiesis, there was a trend toward lower levels of donor chimerism and 3/15 primary recipients did not survive transplantation. Importantly, defects in reconstitution obtained with STAT5ab^null/null^ FL cells were consistent with prior work showing declines in T and B lymphocytes (**Fig. 6B**). As expected, Gab2 deficiency alone did not have any impact upon hematopoietic reconstitution in the absence of a competitor. Differences between STAT5ab^null/null^ and Gab2^−/−^STAT5ab^null/null^ were not significant (P = 0.383; Mann-Whitney U test). Even in the complete absence of STAT5 and Gab2, no additional declines were observed in steady-state hematopoiesis in recipients of double mutant FL transplant relative to STAT5ab^null/null^ alone.

**Figure 7:**
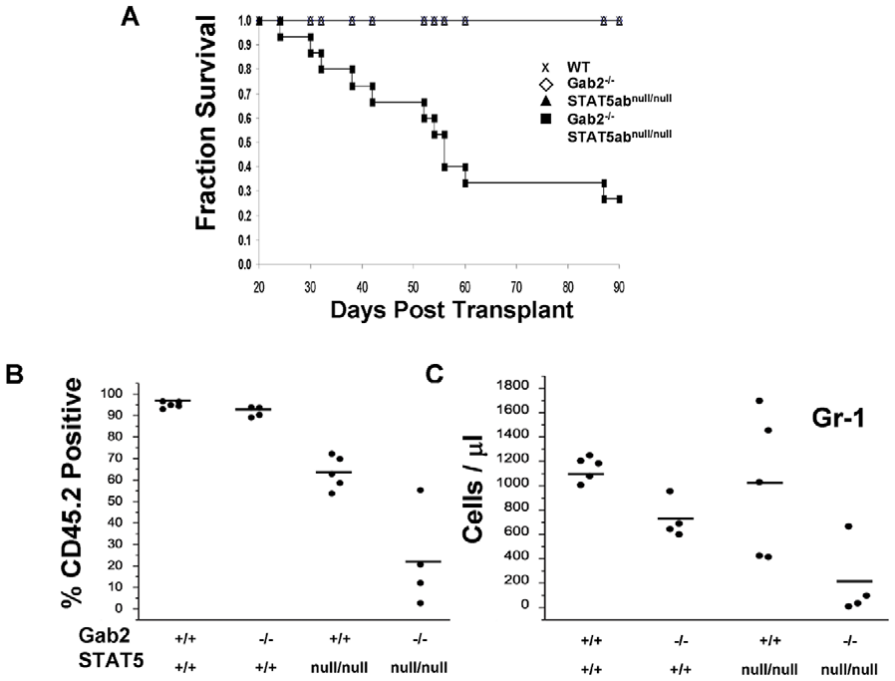
STAT5 and Gab2 double mutant fetal liver HSCs are defective in repopulation of secondary recipients. At 14 weeks following transplant, 3 mice from each group were euthanized from the cohorts of Fig. 6 (Wild-type, Gab2^−/−^, STAT5ab^null/null^, and 3 sets of Gab2^−/−^STAT5ab^null/null^). BM cells were pooled from these primary recipient mice and transplanted into lethally-irradiated secondary CD45.1 hosts at a ratio of 1 donor to 5 recipient mice. **A.** Overall mouse survival was monitored on daily basis for WT N = 5; Gab2^−/−^ N = 4; STAT5ab^null/null^ N = 5; and Gab2^−/−^STAT5ab^null/null^ N = 15. **B.** Donor engraftment in the peripheral blood of secondary transplanted mice for four combinations of STAT5 and Gab2 genotype that survived past approximately 12 weeks for WT N = 5; Gab2^−/−^ N = 4; STAT5ab^null/null^ N = 5; and Gab2^−/−^STAT5ab^null/null^ N = 4. **C.** The absolute number of Gr-1 positive cells was determined by antibody staining and flow cytometry combined with the total white blood cell count per µl of whole blood. Horizontal bars indicate the average for all mice analyzed per group.

Since primary FL transplant cannot measure true HSC self-renewal potential, we collected BM cells from primary hosts and performed BM transplant into lethally-irradiated secondary hosts. Strikingly, 11 out of 15 secondary recipients of Gab2^−/−^STAT5ab^null/null^ FL cells did not survive transplantation up to 12 weeks later, indicating a severe decrease in HSC self-renewal capacity (**Fig. 7A**). Following 12 weeks of reconstitution of the secondary hosts, the surviving recipients of Gab2^−/−^STAT5ab^null/null^ BM were analyzed for donor chimerism along with all of the surviving control mice from the other three groups (**Fig. 7B**). The data show gating for the percentage of donor contribution of CD45.2 engraftment. The donor engraftment was significantly reduced by STAT5ab^null/null^ Gab2^−/−^ genotype (P = 0.004; 2-way ANOVA). Differences between STAT5ab^null/null^ and Gab2^−/−^STAT5ab^null/null^ was moderately significant (P = 0.032; Mann-Whitney U test). These analyses found extremely low donor contribution to the secondary hosts, consistent with graft failure associated with HSC self-renewal deficiency. Accordingly, the absolute number of Gr-1^+^ myeloid cells, a good indicator of real-time hematopoiesis due to the short-half life of neutrophils, was reduced to very low levels when both STAT5 and Gab2 were absent (**Fig. 7C**).

## Discussion

Recent understanding of the impact of cytokine signaling pathways on HSC homeostasis is beginning to show positive and negative effects depending on the pathway and degree of activation. In particular, activation of the PI3-kinase/Akt/mTOR or the Akt/Foxo pathways can have negative consequences on HSC. Foxo3a is pro-apoptotic and must be held in check. Mice lacking PTEN[30], TSC[31], or Foxos[32], [33] have increased HSC proliferation, reduced survival, and loss of repopulating function. Reports of mutant mice lacking Caspase-3[34] or Foxo3a-mediated loss of negative feedback by Spred2[35] demonstrate that Erk pathway activation can also be detrimental to HSC. In contrast, steady-state levels of STAT5 activation positively regulate HSC homeostasis and repopulating function[36].

We have previously reported studies of STAT5 or Gab2 knockout mice and characterized their individual role in hematopoiesis. STAT5 deficiency resulted in severe HSC level defects and was only modestly required in CFU-C[29]. Gab2 was critical for committed CFU-C and multipotent progenitor level response to hematopoietic cytokines and for long-term multilineage competitive repopulating ability of HSC[26]. Gab2 may lie upstream or downstream of STAT5 to promote hematopoiesis or it may function solely through ERK or AKT. Therefore, the studies in this report were designed to identify new Gab2-dependent HSC functions and to determine whether regulation of HSC activity by Gab2 could be accounted for solely by STAT5 in a linear signaling pathway. To do this, the effect of combined deficiency of STAT5 and Gab2 on hematopoiesis was tested following generation of compound mutant mice.

The defects observed in compound mutant mice were informative regarding the levels of hematopoiesis where STAT5 and Gab2 play non-redundant roles and the defects uncovered new functions for Gab2 that were not previously recognized. Using 2-way ANOVA analysis, strong evidence was obtained for non-redundant functions of STAT5 and Gab2 during hematopoiesis. We found that combined deficiency had no additional impact upon peripheral blood hematology or BM cellularity in heterozygous STAT5 mutants. However, combined deficiency resulted in declines in CFU-C and long-term repopulating activity relative to STAT5 or Gab2 deficiency alone. These defects were consistent with the reduced *in vitro* cytokine response, indicative of reduced expansion and differentiation.

Newborn engraftment in the absence of myeloablation to clear the niche and promote hematopoietic engraftment tests the ability of host HSC to compete with donor HSC for engraftment under very stringent conditions that are biased against the donor graft. In this setting, loss of one allele of host STAT5 was sufficient to promote donor engraftment and full deletion of Gab2 alone in the host permitted significant donor reconstitution. Interestingly, the observed defects for Gab2^−/−^STAT5ab^+/null^ mice were greater than predicted for additive. It should also be pointed out that secondary transplantation showed roughly equivalent levels of reconstitution from primary recipients with single or compound mutants. Therefore, the increased donor chimerism associated with non-ablated engraftment may be more associated with selection at the BM multipotent progenitor fraction than with the HSC.

Since in prior studies, Gab2 deficiency was not associated with defects in HSC self-renewal, we wanted to explore whether evidence for Gab2 participation in signaling at the HSC level might be evident in the complete absence of STAT5. STAT5 is important for HSC self-renewal and promotes competitive repopulation ability[37]. Using serial transplantation, we were able to uncover important roles for Gab2 in HSC signaling. In our prior work[37], STAT5-deficient HSC caused death in quaternary transplants. However, Gab2-deficient HSC permitted tertiary transplantation (data not shown[26]). The results of serial transplant using FL cells from mice completely lacking STAT5 and Gab2 were very striking. The combined defects in secondary transplant reported here indicate that HSC depletion occurs following the first transplant, despite reasonable engraftment of the granulocytic lineage. This type of secondary transplantation failure is comparable to a recent report of E12.5 FL cells lacking Wnt3a[38]. Therefore, Gab2 can play an important role in normal HSC self-renewal in a novel manner by facilitating the role of STAT5 in HSC function.

To better understand why the HSC number was decreased in the FL of STAT5/Gab2 double mutant mice, flow cytometry assay for apoptosis was performed. The results of this assay indicate that reduced survival can account for the reduction in HSC number. STAT5 is known to be an important regulator of survival signaling and Gab2 facilitates Akt-mediated survival signals. However, the striking reduction in survival signaling is novel and suggests either direct effects between downstream targets mediated by STAT5 and PI3-K pathways or cross-talk between these pathways could be critical for optimal survival signaling. In the context of competitive repopulation and self-renewal activities, additional studies will be required to identify whether defective engraftment is due to additional loss of proliferative or homing potential. Limitations in the number of double knockout FL that could be obtained prohibited assays requiring large HSC numbers. Conditional deletion of STAT5 in adult BM may provide a tool for future studies.

The unique roles for STAT5/Gab2 interaction during non-ablated engraftment indicate important function in generation and maintenance of HSC. The important role of STAT5 in many aspects of HSC function is now clear. However the mechanisms of action of STAT5 remain largely undefined. Defining and targeting interactive signaling nodes has become important area of research for stem cell transplantation and eradicating leukemic stem cells. The finding that Gab2 can have STAT5-independent functions in HSC indicates that activation of the PI3-K or MAPK pathways could be beneficial for steady-state HSC function. In addition, these Gab2 functions appear to synergize with STAT5 in the most critical aspects of HSC function, namely self-renewal as assayed by serial transplantation. A better understanding of the importance of STAT5/Gab2 signals may lead to new approaches for non-ablative HSC transplantation or therapeutics for hematologic malignancies where HSC survival and self-renewal are dysregulated.

## Supporting Information

Figure S1 No changes in peripheral blood hematology or LT-HSC from single or compound mutant adult mice. A. Steady-state peripheral hematology was determined by total WBC counts using a Coulter counter combined with flow cytometry analysis for cells staining positive for lineage markers. Lineage antibodies to Gr-1, Mac-1, B220, Ter119, CD4, and CD8 were used. No significant differences were observed for any hematologic parameter in WT (N = 11), STAT5ab+/null (N = 11), Gab2−/− (N = 11), or Gab2−/−STAT5ab+/null (N = 13) mice. B. BM cells were collected and stained with lineage antibodies, combined with the combination of Sca-1, c-Kit, Flk2 (Flt3), and CD48 and the values plotted represent the average number of cells per adult mouse (2 tibias and 2 femurs) for WT (N = 8), STAT5ab+/null (N = 9), Gab2−/− (N = 9), or Gab2−/−STAT5ab+/null (N = 9) mice.
(4.19 MB TIF)

Figure S2 Flow cytometry gating for analysis of FL KLS and KLS CD150+CD48− populations. FL cells were collected from E14.5 embryos and stained with antibodies to determine the percentage of LT-HSC present. Initial gating was performed using 7-AAD to exclude dead FL cells. Within the 7-AAD negative/low fraction, the cells were gated on forward and side scatter to also collect the viable fraction. The lineage-negative fraction was then gated for expression of KLS cells. As an additional measure of the LT-HSC fraction within the KLS gate, additional gating for CD150+CD48− cells was performed as shown. Note that the numbers shown in the right two columns are the frequencies of the total population, not just the population shown.
(4.02 MB TIF)
