# Supplementary figures and images for: Gab2 Promotes Hematopoietic Stem Cell Maintenance and Self-Renewal Synergistically with STAT5
Source: PLoS One. 2010 Feb 10;5(2):e9152. doi: 10.1371/journal.pone.0009152 (PMC2818849; doi:10.1371/journal.pone.0009152)

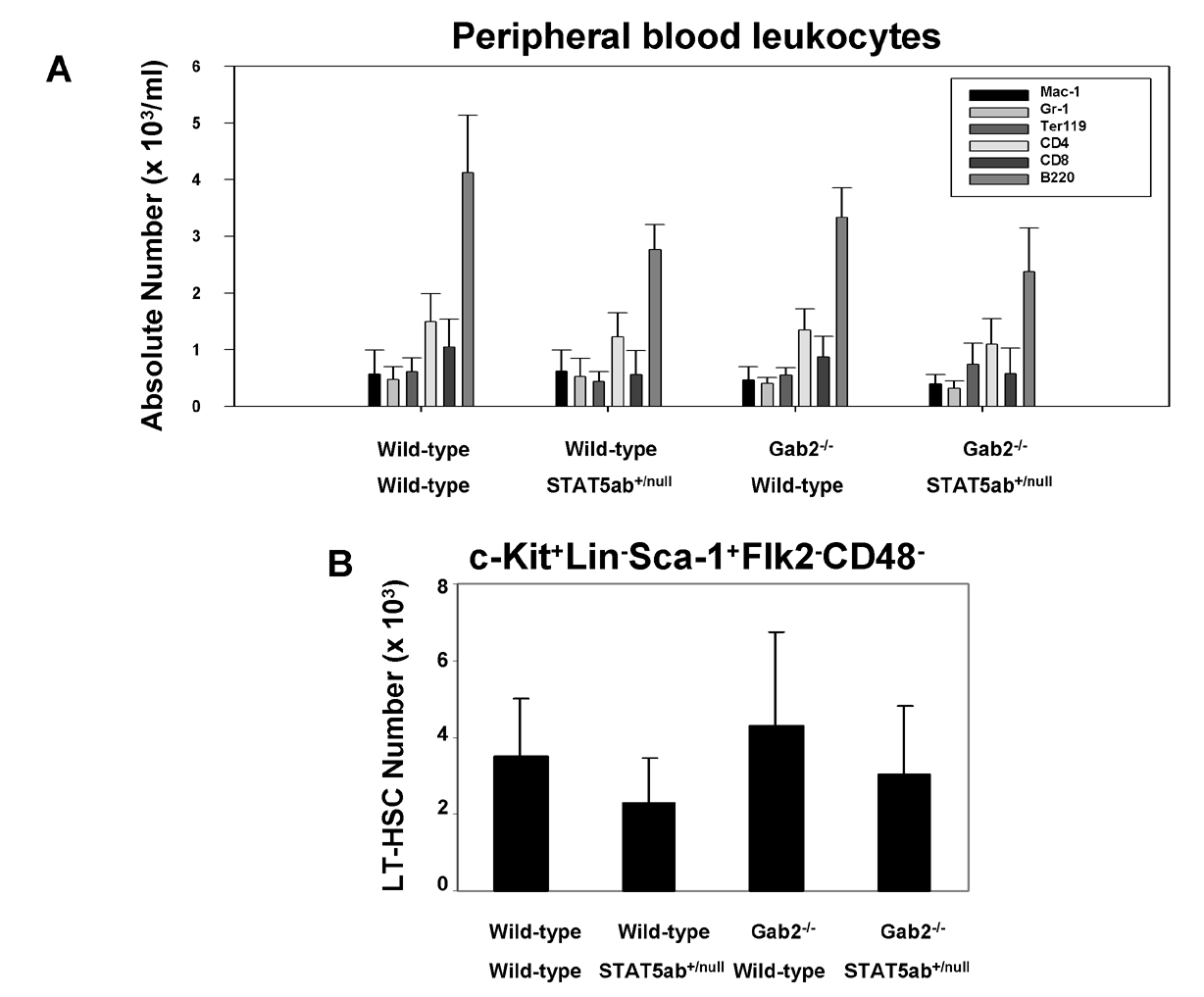

Supplement: Figure S1 — No changes in peripheral blood hematology or LT-HSC from single or compound mutant adult mice. A. Steady-state peripheral hematology was determined by total WBC counts using a Coulter counter combined with flow cytometry analysis for cells staining positive for lineage markers. Lineage antibodies to Gr-1, Mac-1, B220, Ter119, CD4, and CD8 were used. No significant differences were observed for any hematologic parameter in WT (N = 11), STAT5ab+/null (N = 11), Gab2−/− (N = 11), or Gab2−/−STAT5ab+/null (N = 13) mice. B. BM cells were collected and stained with lineage antibodies, combined with the combination of Sca-1, c-Kit, Flk2 (Flt3), and CD48 and the values plotted represent the average number of cells per adult mouse (2 tibias and 2 femurs) for WT (N = 8), STAT5ab+/null (N = 9), Gab2−/− (N = 9), or Gab2−/−STAT5ab+/null (N = 9) mice. (4.19 MB TIF) [file pone.0009152.s001.tif]

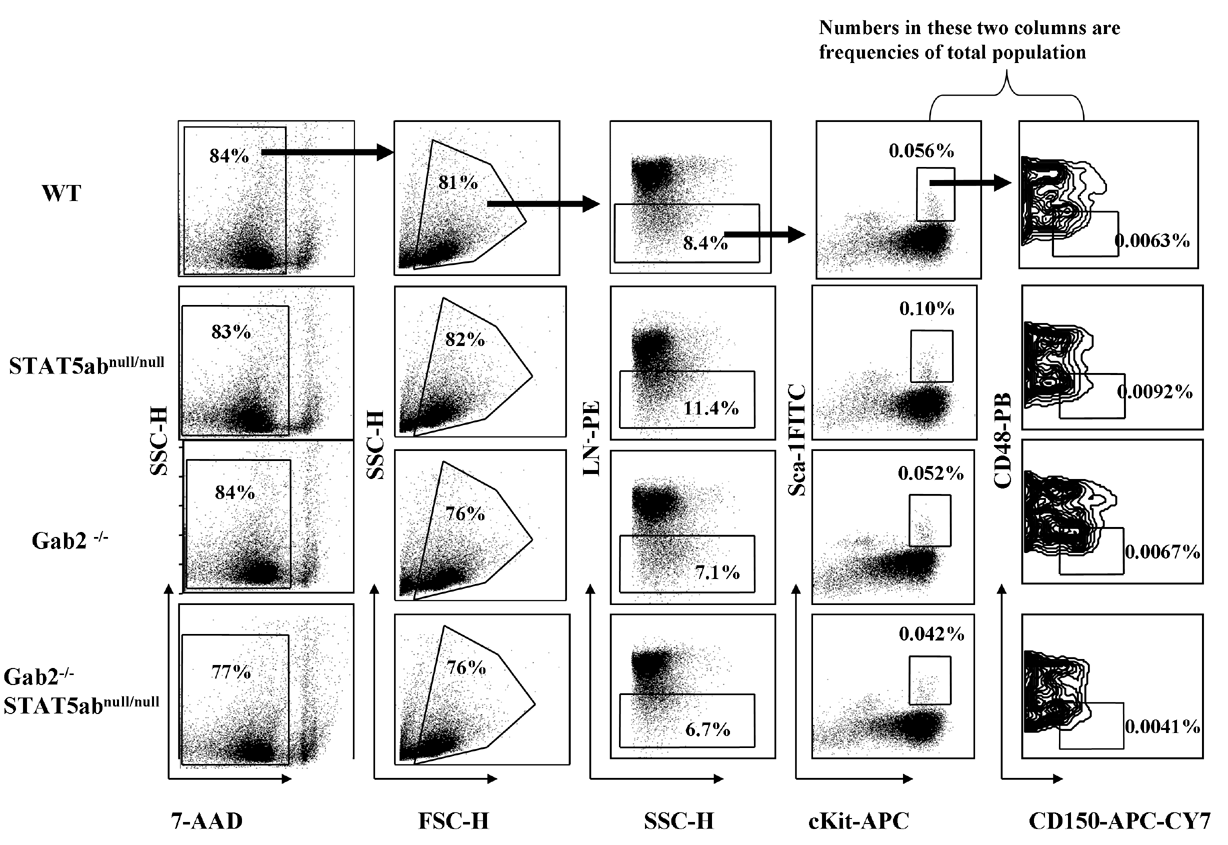

Supplement: Figure S2 — Flow cytometry gating for analysis of FL KLS and KLS CD150+CD48− populations. FL cells were collected from E14.5 embryos and stained with antibodies to determine the percentage of LT-HSC present. Initial gating was performed using 7-AAD to exclude dead FL cells. Within the 7-AAD negative/low fraction, the cells were gated on forward and side scatter to also collect the viable fraction. The lineage-negative fraction was then gated for expression of KLS cells. As an additional measure of the LT-HSC fraction within the KLS gate, additional gating for CD150+CD48− cells was performed as shown. Note that the numbers shown in the right two columns are the frequencies of the total population, not just the population shown. (4.02 MB TIF) [file pone.0009152.s002.tif]
